# Identifying the Soybean microRNAs Related to *Phytophthora sojae* Based on RNA Sequencing and Bioinformatics Analysis

**DOI:** 10.3390/ijms24108546

**Published:** 2023-05-10

**Authors:** Zhanguo Zhang, Song Jin, Huilin Tian, Zhihao Wang, Rui Jiang, Chunyan Liu, Dawei Xin, Xiaoxia Wu, Qingshan Chen, Rongsheng Zhu

**Affiliations:** 1 College of Arts and Sciences, Northeast Agricultural University, Harbin 150030, China; 2 National Research Center of Soybean Engineering and Technology, Harbin 150030, China; 3 College of Agriculture, Northeast Agricultural University, Harbin 150030, China; 4 College of Life Sciences, Northeast Agricultural University, Harbin 150030, China

**Keywords:** *Phytophthora sojae* (*P. sojae*), microRNA, quantitative real-time PCR (qRT-PCR), transcription factor, evolution

## Abstract

Phytophthora root rot in soybeans is caused by a pathogen called *Phytophthora sojae* (*P. sojae*), which results in a significant decrease in soybean production within affected regions. MicroRNAs (miRNAs) are a class of small non-coding RNA molecules that play a key post-transcriptional regulatory role in eukaryotes. In this paper, the miRNAs that respond to *P. sojae* were analyzed from the gene level to complement the study of molecular resistance mechanisms in soybean. The study utilized high-throughput sequencing of soybean data to predict miRNAs that respond to *P. sojae*, analyze their specific functions, and verify regulatory relationships using qRT-PCR. The results showed that the miRNAs in soybean respond to *P. sojae* infection. MiRNAs can be transcribed independently, suggesting the presence of transcription factor binding sites in the promoter regions. Additionally, we performed an evolutionary analysis on conserved miRNAs that respond to *P. sojae*. Finally, we investigated the regulatory relationships among miRNAs, genes, and transcription factors, and identified five regulatory patterns. These findings lay the groundwork for future studies on the evolution of miRNAs responsive to *P. sojae*.

## 1. Introduction

Soybean is native to China and is an important food crop with high edible, economic, and medicinal value. However, soybean production has a low yield, and improving soybean disease resistance and environmental adaptability would be a direct way to increase soybean production. Soybean *Phytophthora* root rot, one of the most serious soybean diseases, has been reported in 12 countries, including the United States, and is a global soybean disease [1].

Currently, the area where *Phytophthora* root rot is infecting soybean production is increasing [2]. Since Johal et al. cloned the *Hm1* disease-resistant gene from maize using transposon tagging technology in 1992 [3], researchers in various countries have successively obtained more than 70 disease-resistant genes from 10 species of plants, including rice. In 2018, Qun Cheng et al. found and verified that the bHLH transcription factor GmPIB1 promoted resistance to *Phytophthora sojae* [4]. Wei Li et al. cloned a *Phytophthora sojae* resistance gene, RpsYD29 GmZFP03, which encodes a zinc finger protein-type transcription factor that exhibits resistance to *P. sojae* at the molecular level by targeting the promoters of two SOD1 genes and activating their expression [5]. James Wong et al. demonstrated the involvement of miR393 and miR166 in the basic defense against *Phytophthora sojae* [6]. Chen et al. confirmed that the dirigent (DIR) domain protein GmDRR1 (Glycine max Disease Resistance Response 1) in Suinong 10 soybean exhibits resistance to Phytophthora root rot in soybean [7].

MicroRNAs (miRNAs) are small, non-coding RNAs that play crucial roles in plant stress responses. Numerous studies have been conducted on miRNA functions in recent years, revealing their post-transcriptional regulatory role in various aspects of organism growth, development, nutrition, metabolism, and stress resistance. In 2002, three laboratories (Carrington, Chen, and Bartel) used RNA cloning to obtain over 100 endogenous miRNAs from Arabidopsis; later on, the Zamore laboratory identified 280 miRNAs, including the first plant miRNA known as miR171 [8]. Multiple studies have been conducted on miRNA functions in recent years, and it has been discovered that miRNAs play a post-transcriptional regulatory role in several aspects of organism growth, development, nutrition, metabolism, and stress resistance. In 2012, Li et al. discovered that miRNAs played an important regulatory role in Soybean cyst nematodes (SCN) [9]. In animals, miR-21 [10,11], miR-24 [12], miR-375 [13], miR-196a [14], miR-132 [15], and a large number of other miRNAs have been found to be associated with diseases—discoveries which provide new technical directions for the treatment of animal diseases. Guo et al. initially demonstrated that miRNAs had a regulatory role when affecting *P. sojae* infestation [16].

MiRNAs exercise their functions by regulating the expression of target genes and regulating their own synthesis [17]. Yan et al. demonstrated that MiR172 is associated with soybean nodulation, and that miR172 is regulated by miR156, which in turn affects the regulation of AP2 by miR172, and thus soybean modulate [17]. Ye et al. predicted two regulatory mechanisms: TF-miRNA-mRNA and lncRNA-miRNA-mRNA [18]. Two regulators exist in organisms, miRNA and transcription factors, both of which can regulate genes and each other, and contribute to the stability of life in biological systems.

However, to fully comprehend their precise interactions, further experiments are required. The utilization of miRNAs for studying soybean resistance to *P. sojae* can aid in understanding the mechanism of plant disease resistance genes and improving plant resistance. In this study, we sequenced the expression profile of the *P. sojae*-resistant soybean variety SN10 at different times after inoculation with virus race one, and conducted large-scale reverse prediction of *P. sojae*-related miRNAs using genomic differential expression data. We identified twelve highly reliable miRNAs involved in the plant’s defense against *P. sojae,* and verified 30 valuable miRNA-target pairs through q-PCR experiments. Additionally, we predicted twelve genes that may be involved in soybean resistance to Phytophthora root rot. Finally, we summarized the regulatory modes of five disease response miRNAs along with their target transcription factors in plants.

## 2. Results

### 2.1. Expression Profile Sequencing Analysis and Differential Expression Analysis

To study the genes associated with *Phytophthora* root rot resistance in soybean, we sequenced the Suinong No.10 soybean line expression profile. The sequencing samples were SCKL (Sample Control Check Leaf), SML (Sample Medium Leaf), STL (Sample Treatment Leaf) 0.5, STL2, and STL4. A total of 33,683 gene fragments were detected in the expression profile sequencing data of the five samples (Supplementary Materials Table S1). Differently expressed genes were screened for those with significant expression changes between samples. Six groups of SML-STL0.5, SML-STL2, SML-STL4, STL0.5-STL2, STL0.5–STL4, and STL2–STL4 samples were compared, and the number of genes with differential expression was 6187, 309, 756, 6101, 6587, and 1685, respectively, of which there were 8958 common differently expressed genes (Supplementary Materials Table S2). The genes with expression changes between the two SCKL-SML samples were screened, and 1947 genes that may be related to the scratching and inoculation of an empty medium were obtained. Of them, 1557 were also screened in the previous six groups. Excluding the 1557 genes that may be related to the scratching and inoculation of an empty medium, 7401 reliable differently-expressed genes were obtained; that is, 7401 *P. sojae* resistance genes were used as the *P. sojae* associated gene set (Supplementary Materials Table S3).

### 2.2. Identification of Target Genes and Screening of P. sojae-Related miRNAs

**Figure 1:**
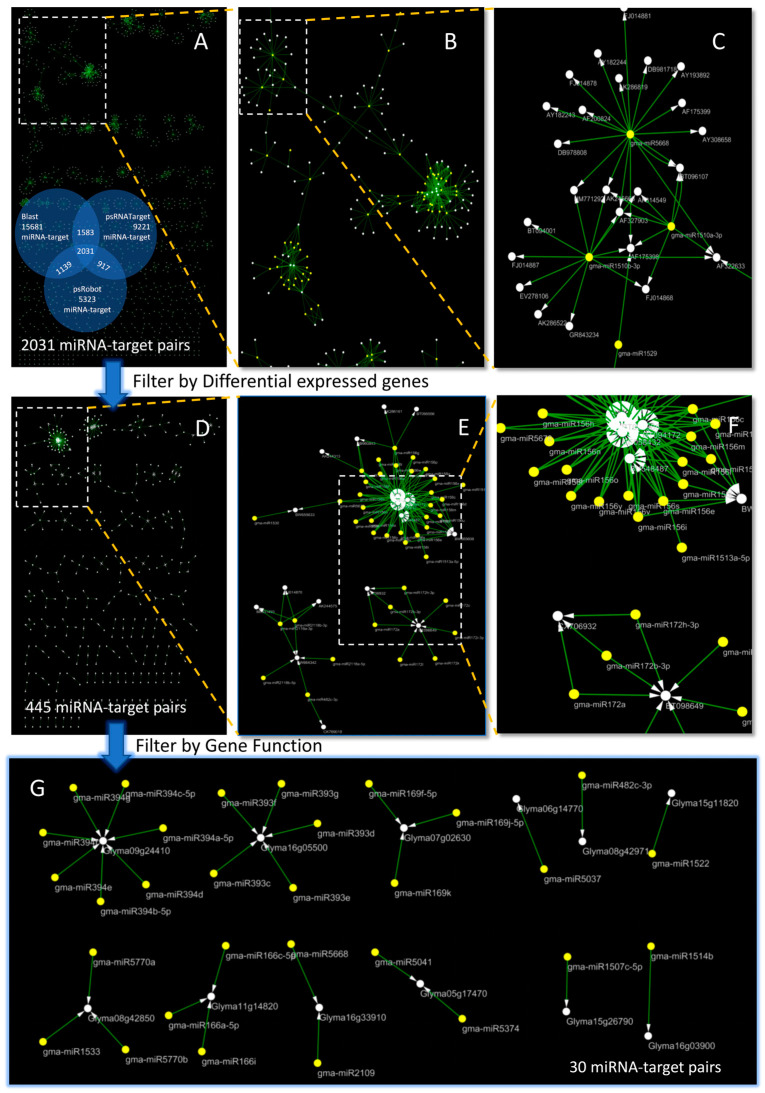
Results of systematic identification of soybean miRNA’s resistance to *P. sojae*. (**A**) Visualization of the identified 2031 miRNA-target relationship results; The Venn diagram shows the 2031 relationship pairs identified by the three methods. (**B**,**C**) indicate the gradual amplification of (**A**); The yellow circles represent miRNA and the white circles represent gene; The arrow represents the targeting relationship between miRNA and Gene ((**E**–**G**) are the same). (**D**) 2031 miRNA-target pairs were filtered out by differently expressed genes, and 445 miRNA-target pairs were obtained. (**E**,**F**) are the gradual amplification of (**D**). (**G**) According to gene function screening, 30 pairs of miRNA-target relationships were finally obtained.

To predict the miRNA-target relationship, we used BLAST, psRNATarget, psRobot 3 prediction software, and miRNA from the 19.0 version of miRBase. A total of 555 miRNAs were downloaded, and the identified gene target set selected 33,683 spliced sequence fragments in the expression profile sequencing. A total of 15,681 miRNA-target relationships were predicted by BLAST (Supplementary Materials Table S4). When using BLAST to predict the miRNA target, all the results that met the conditions were obtained by relaxing the conditions without violating the principle of miRNA target genes. However, due to the optimal principle of BLAST, a large amount of redundant data was also generated. Meanwhile, psRNATarget and psRobot predicted 9221 and 5323 miRNA-target relationships, respectively (Supplementary Materials Tables S5 and S6). According to the name and location of the miRNA-target gene, the results predicted by the three software programs were overlapped, and 2031 miRNA-target relationships were obtained, including 987 genes and 454 miRNAs (Figure 1A–C; Supplementary Materials Table S7).

**Figure 2:**
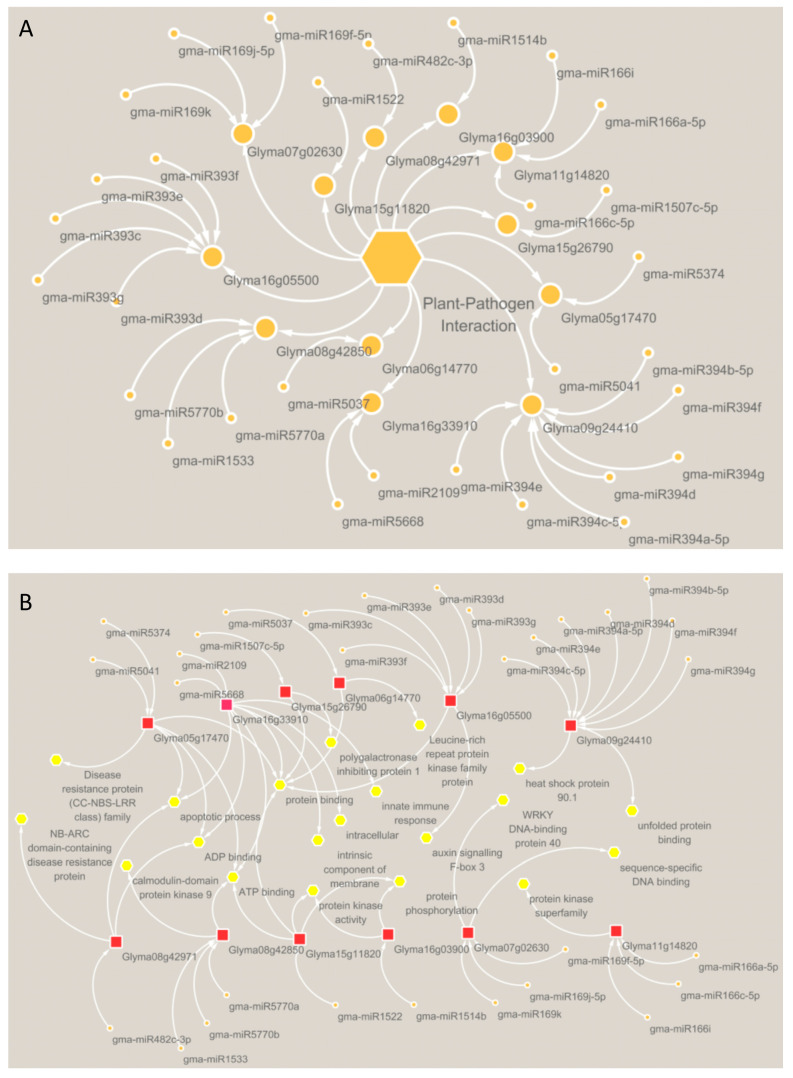
Relation networks of 30 miRNA-target pairs. (**A**) The pathway of miRNA-target pairs was annotated to indicate the relationship between the miRNA and the target. Small orange circles represent miRNA; big orange circles represent gene; the orange hexagon represents plant–pathogen interaction; circle and circle represent the target relationship; the arrows between the hexagon and the large circles represent the genes and annotated pathway. (**B**) Detailed functional annotation of the miRNA-target pairs pathogen. Orange circles represent miRNA; the red rectangles represent the gene; yellow circles represent pathways; the arrows between the orange circle and red rectangle represent the targeting relationship; the arrows between the yellow circles and the red rectangle represent the pathways to which the gene is annotated.

To clarify whether the miRNA-target relationship was induced by *P. sojae*, 233 differently expressed genes, regulated by miRNAs, were screened from 7401 genes with expression changes. These 233 genes correspond to 256 miRNAs and 445 miRNA-target relationships (Figure 1D–F, Supplementary Materials Table S8). Through the annotation information provided by GO, KEGG, and Soybase, we finally screened out 30 pairs of miRNA-target relationships related to soybean *P. sojae* resistance (Figure 1G and Figure 2A). The KEGG and GO annotation information for 30 pairs of miRNA-target relationships associated with soybean resistance to *P. sojae* are shown in Figure 2B.

### 2.3. Distribution of Soybean Resistance-Related miRNAs and Target Genes on Chromosomes

**Figure 3:**
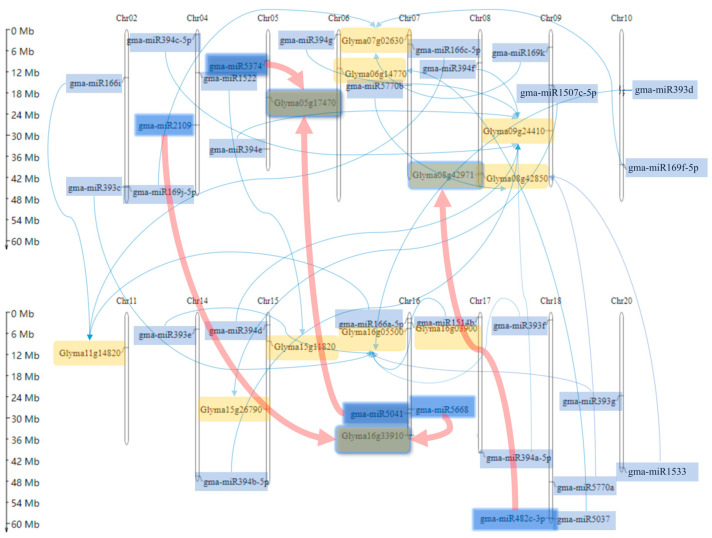
A total of 30 pairs of miRNA-target genes on the distribution of 20 chromosomes in soybean, and the miRNA-target interaction; the blue arrows represent the targeting relationship of the miRNA target; the red arrows represent the miRNA-target pairs that have been verified by q-PCR experiments; the yellow rectangles represent the target gene in the miRNA target; the blue rectangles represent miRNA in the miRNA target.

Thirty pairs of miRNA-target pairs related to *P. sojae* were mapped on 20 soybean chromosomes, and the interaction relationship was marked (Figure 3). Among them, a total of 30 miRNAs and 12 target genes were distributed on 15 chromosomes. There were no miRNAs or target genes in the 30 relationship pairs on the Chr01, Chr03, Chr12, Chr13, or Chr19 chromosomes. Genes and miRNAs related to *P. sojae* resistance were widely distributed. Most of the chromosomes had genes involved in the regulation of soybean resistance to *P. sojae*. Only the target gene Glyma11g14820 on chromosome 11 was regulated by three miRNAs. Furthermore, 7 chromosomes had only miRNA or a combination of miRNA and target genes. The targeted interaction of miRNAs was more likely to act on genes located in different miRNAs chromosomes. Among the three gene-related pairs, miRNAs and target genes were located on the same chromosome and the remaining relationship pairs were located on different chromosomes (Figure 3).

### 2.4. Verification of Soybean Resistance-Related miRNA and Target Gene Expression

**Figure 4:**
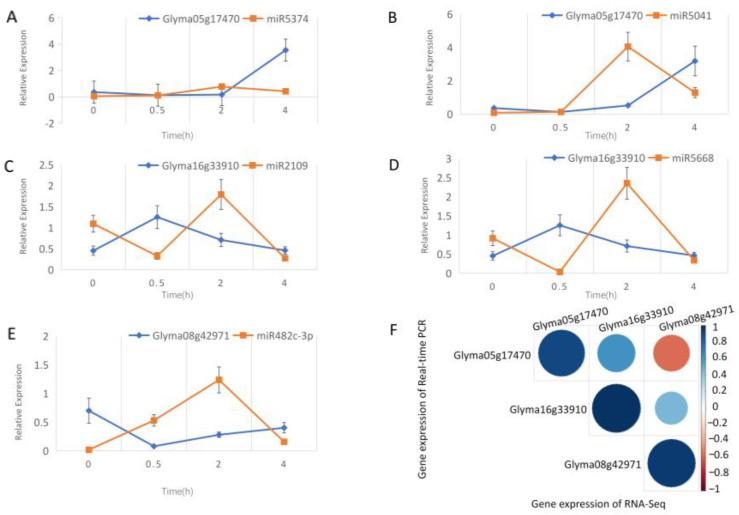
Expression analysis of miRNA and target gene by qRT-PCR and RNA-Seq. (**A**) Expression analysis of Glyma05g17470 and gma-miR5374. The X-axis represents the sample corresponding to the inoculation time, and the y-axis represents the relative expression ((**B**–**E**) are the same). (**B**) Expression analysis of Glyma05g17470 and gma-miR5041. (**C**) Expression analysis of Glyma16g33910 and gma-miR2109. (**D**) Expression analysis of Glyma16g33910 and gma-miR5668. (**E**) Expression analysis of Glyma08g42971 and gma-miR482c-3p. (**F**) Correlation analysis between the gene expression of qRT-PCR and the gene expression of RNA-seq for each gene. The size of the circles represents the degree of correlation. The stronger the correlation, the larger the circle. The color represents the value of the correlation; the closer the correlation is to 1, the color is dark blue; the closer the correlation is to −1, the color is dark red; when the correlation is equal to 0, the color is white.

Glyma16g33910, Glyma05g17470, and Glyma08g42971 are disease-related genes in plant-pathogen interaction. Glyma08g42971 and Glyma05g17470 played an important role in resisting biological stress caused by fungi and soybean nodulation, respectively [19,20]. Glyma16g33910 was identified as a nucleotide-binding site and leucine-rich repeat (NBSLRR) gene, which may be related to soybean disease resistance [21]. Glyma16g33910 is regulated by gma-miR2109 and gma-miR5668, and Glyma05g17470 is regulated by gma-miR5041 and gma-miR5374. Four pairs of miRNA targets showed a significant negative correlation. Glyma08g42971 was regulated by gma-miR482c-3p, which showed a negative correlation with the miRNA target except over a 0.5–2 h period. Following qRT-PCR verification, it was confirmed that the five pairs of miRNA-target regulatory relationships in the plant-pathogen interaction pathway were related to *P. sojae* resistance (Figure 4A–E).

The correlation between the gene expression of qRT-PCR and the gene expression of RNA-seq was analyzed for each gene. Both results for the same genes were highly correlated. Glyma16g33910 and Glyma05g17470, and Glyma08g42971 and Glyma05g17470 were positively correlated in expression, while Glyma08g42971 and Glyma05g17470 were negatively correlated in expression (Figure 4F).

### 2.5. Analysis of Action Mode

**Figure 5:**
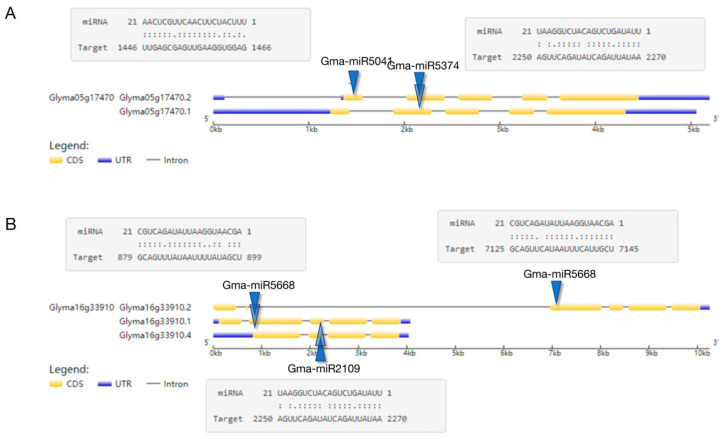
Analysis of the action mode between miRNA and target genes. (**A**) Multiple miRNAs act on one gene: Gma-miR5041 and Gma-miR5374 act together on the CDS region of Glyma05g17470.2; The blue triangles represent the position of the miRNA target on the gene ((**B**) is the same). (**B**) One miRNA acts on multiple genes: Gma-miR5668 affects the CDS regions of the Glyma16g33910.1, Glyma16g33910.2, and Glyma16g33910.4 transcripts in the Glyma16g33910 gene, and Gma-mir2109 acts on the CDS region of the Glyma16g33910.1 and Glyma16g33910.4 transcripts.

By mapping the miRNA target gene position, it was found that one miRNA can act on multiple target genes and that multiple miRNAs can act on just one target gene. To better understand the function of miRNA in dynamic processes, it is important to identify the action site on the gene and investigate how miRNA targets operate. Additionally, examining the status of detailed action sites can provide valuable insights into miRNA’s mode of action. For example, Gma-miR5041 and Gma-miR5374 act together on the CDS region of Glyma05g17470.2 (Figure 5A); Gma-miR5668 affects the CDS regions of the Glyma16g33910.1, Glyma16g33910.2, and Glyma16g33910.4 transcripts in the Glyma16g33910 gene; and Gma-mir2109 acts on the CDS region of the Glyma16g33910.1 and Glyma16g33910.4 transcripts (Figure 5B).

### 2.6. Evolutionary Signatures of Soybean miRNA and Target Genes

**Figure 6:**
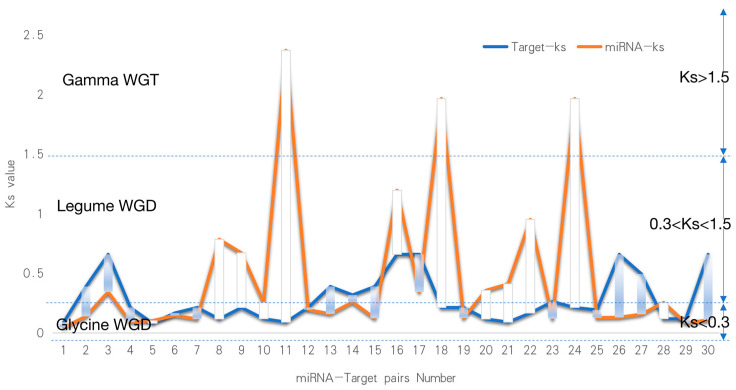
The Ks values for 30 disease-resistant miRNA-target pairs; the ks value of soybean can be divided into three stages: 0 < ks < 0.3 represents the latest whole Genome Duplication stage; 0.3 < ks < 1.5 represents the second whole genome replication period; ks > 1.5 is the farthest doubling event for dicotyledonous plant differentiation.

**Figure 7:**
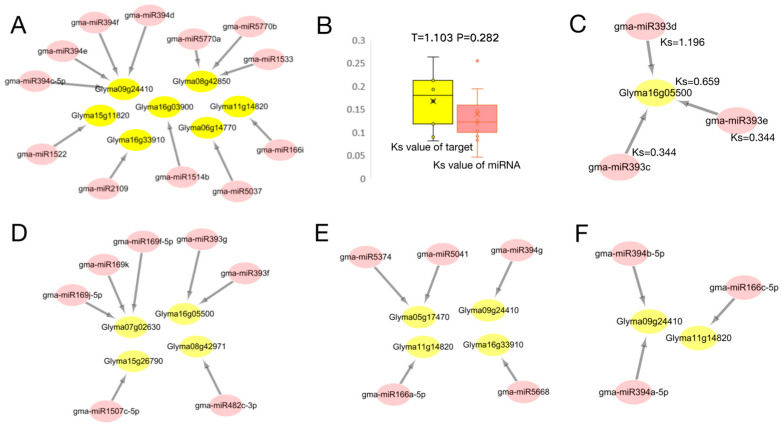
Evolutionary analysis of miRNA and target genes based on Ks values and divided into 5 categories. (**A**) Both miRNA and targets appeared in the soybean Glycine WGD period (miRNA Ks < 0.3. target Ks < 0.3); The pink ellipses represent miRNA, and the yellow ellipses represent genes; The grey arrows represent the targeting relationship ((**C**–**F**) are the same). (**B**) The boxplot of the Ks value of miRNA in Glycine WGD and the targets in Glycine WGD (*p* > 0.05). (**C**) MiRNA in Legume WGD and the targets in Legume WGD (0.3 < miRNA Ks < 1.5, 0.3 < target Ks < 1.5). (**D**) MiRNA in Glycine WGD and the targets in Legume WGD (miRNA Ks < 0.3, 0.3 < target Ks < 1.5). (**E**) MiRNA in Legume WGD and the targets in Glycine WGD (0.3 < miRNA Ks < 1.5, target Ks < 0.3). (**F**) MiRNA in Gamma WGT and the targets in Glycine WGD (miRNA Ks > 1.5, target Ks < 0.3).

We calculated the Ks values for 30 disease-resistant miRNA-target pairs and divided them into five classes of miRNA targets based on the Ks values (Figure 6). The Ks value is indicative of the generation time of homologous genes. The whole genome doubling event will produce a large number of homologous genes, which will be reflected in the Ks value. As a consequence, there will be a significant number of homologous gene pairs with similar Ks values [22]. Greater than 60% of the Arabidopsis genome segment is duplicated, and 75% of the soybean gene segment has multiple copies. Studies have shown that soybean’s genome has historically undergone three whole genome duplication events, the first occurring in the ‘Gamma’ period about 130 million years ago (Ks > 1.5), which occurred after the monocotyledonous differentiation and the third in triplication. The two subsequent occurrences were in the “Legume” period (0.3 < Ks < 1.5) and the “Glycine” period (0 < Ks < 0.3) 59 and 13 million years ago, respectively, and the subsequent replications were both diploid [23]. (1) Both miRNA and targets appeared in the soybean Glycine Whole Genome Duplication (WGD) period (miRNA Ks < 0.3. Target Ks < 0.3, Figure 7A), and an independent *T*-test based on miRNA-target Ks values showed consistent miRNA-target evolution (*p* > 0.05, Figure 7B). (2) miRNA was found in Legume WGD and the targets in Legume WGD (0.3 < miRNA Ks < 1.5, 0.3 < target Ks < 1.5, Figure 7C). (3) miRNA was found in Glycine WGD and the targets in Legume WGD (miRNA Ks<0.3, 0.3 < target Ks < 1.5, Figure 7D). (4) miRNA was found in Legume WGD and the targets in Glycine WGD (0.3 < miRNA Ks < 1.5, target Ks < 0.3, Figure 7E). (5) miRNA was found in Gamma WGT and the targets in Glycine WGD (miRNA Ks > 1.5, target Ks < 0.3, Figure 7E). The above five types of targeting relationship, over the three stages of evolution, have a new miRNA which is associated with *P. sojae,* but is only associated with disease-resistant genes in the Legume WGD and Glycine WGD stages. This also illustrates that the evolutionary miRNA is constantly corresponding to gene evolution and evolution, further emphasizing the important role of miRNA in the process of disease resistance.

### 2.7. Mining of Potential Soybean P. sojae-Resistant Genes Based on Identified miRNA

**Figure 8:**
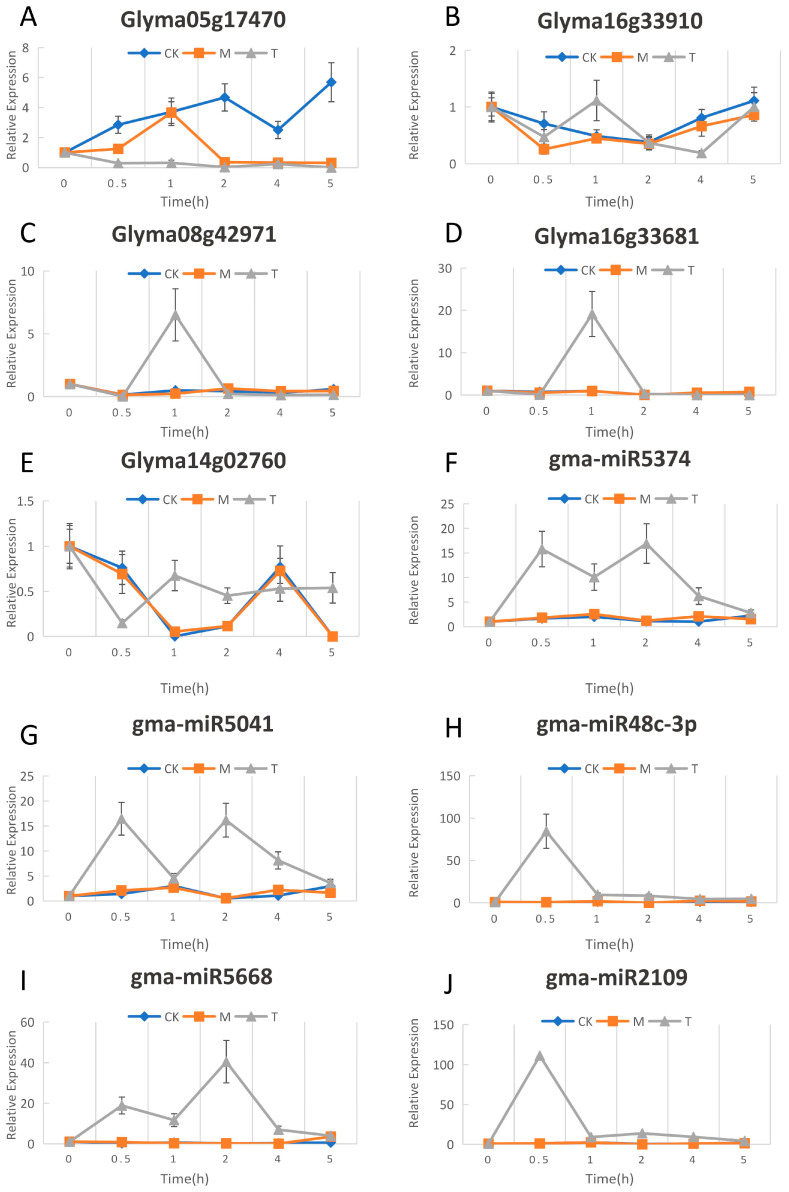
The change curves of the identified target gene and miRNA expression under three treatments; the X-axis represents the sample corresponding to the inoculation time, and the Y-axis represents the relative expression. (**A**–**E**) Glyma05g17470, Glyma16g33910, Glyma08g42971, Glyma16g33681, Glyma14g02760; (**F**–**J**) gma-miR5374, gma-miR5041, gma-miR48c-3p, gma-miR48c-3p, gma-miR2109. The control group was labeled as CK (without any treatment), while M indicated samples vaccinated with an empty medium and T represented samples vaccinated with *P. sojae*.

**Figure 9:**
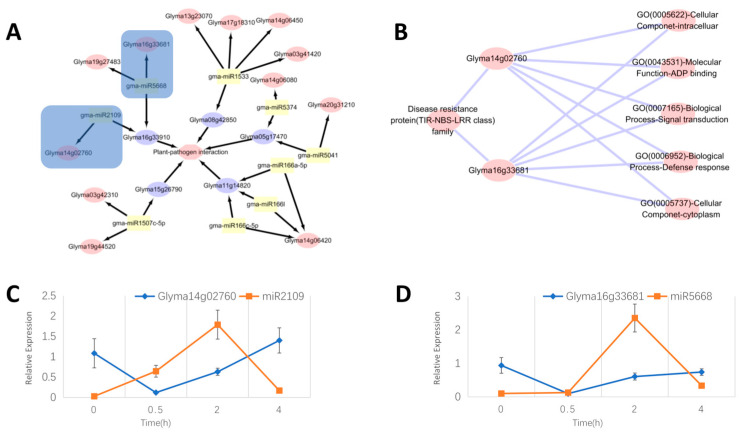
Pathway analysis, function annotation and qRT-PCR result. (**A**) Pathway analysis of genes related to disease resistance. The outer pink ellipses represent the predicted gene that may be associated with *P. Sojae* resistance. The purple ellipses represent the genes directly related to *P. Sojae* resistance identified in this study. The yellow ellipses represent miRNA. The middle pink ellipse represents plant–pathogen interaction. The black arrow represents the targeting relationship. (**B**) Enrichment analysis of target relation in 9A blue box. The purple lines represent gene and annotation relationships. (**C**) Expression analysis of Glyma14g02760 and gma-miR2109. The X-axis represents the sample corresponding to the inoculation time, and the Y axis represents the relative expression ((**D**) is the same). (**D**) Expression analysis of Glyma16g33681 and gma-miR5668.

We compared the expression change plots of genes and miRNAs in the three CK, M, and T samples (CK means without any treatment, M indicates samples vaccinated with an empty medium, T indicates samples vaccinated with *P. sojae*), and found that there are consistent trends between the CK and M gene curves of genes and miRNAs. This indicates that for the resistance gene and the miRNA response to *P. sojae*, scratch and inoculation in the empty medium did not have much effect. However, the gene or miRNA expression change curve of the inoculated *P. sojae* sample was clearly different from the M curve. This further illustrates that these five genes and five miRNAs all respond to infections of *P. sojae* (Figure 8). Pathway analysis revealed that plant–pathogen interaction was mainly affected by the following five genes: Glyma08g42850, Glyma05g17470, Glyma11g14820, Glyma15g26790, and Glyma16g33910. Five genes were affected by the 9 miRNAs, and the 9 miRNAs acted on 12 genes (Figure 9A). These 12 genes may also impact soybean resistance to *P. sojae*. Of the 12 genes, Glyma03g41420, Glyma03g42310, Glyma14g06420, Glyma17g18310, and Glyma19g44520 were the five genes used in this study to identify *P. sojae* resistance and might be unique. Glyma16g33681 was suggested to play an important role in resistance to soybean powdery mildew [24]. The Glyma19g27483 gene was related to isoflavone content in soybean seeds [25]. The expression of the Glyma13g23070 gene was highly regulated when roots were exposed to *P. sojae*, glycine, and aluminum stress, which may be a multifunctional stress resistance gene in soybean [26]. The Glyma14g06450 gene showed tolerance to drought stress in soybean. Glyma14g06080, as one of the two homologous compounds of the DREB2 family, can enhance the life of tobacco seed when DREB2 and the heat shock factor HaHSFA9 from sunflower are overexpressed [27]. It has been reported that the Glyma20g31210 gene is highly expressed in salt tolerance [28]. The Glyma14g02760 gene plays a role in soybean *Phytophthora* root rot [29]. Two pairs of relationships, gma-miR5668 and Glyma16g33681, and gma-miR2109 and Glyma14g02760, were annotated to the disease resistance protein (TIR-NBS-LRR class) family and the notes are the same (Figure 9B). In the qRT-PCR analysis, there were also significant relationships of reciprocal inhibition between miRNAs and targets (Figure 9C,D). It was verified that the 12 genes affected by miRNAs may indeed express resistance to *P. sojae*.

### 2.8. Analysis of the Interaction Patterns of miRNA and Transcription Factor(TF)

**Figure 10:**
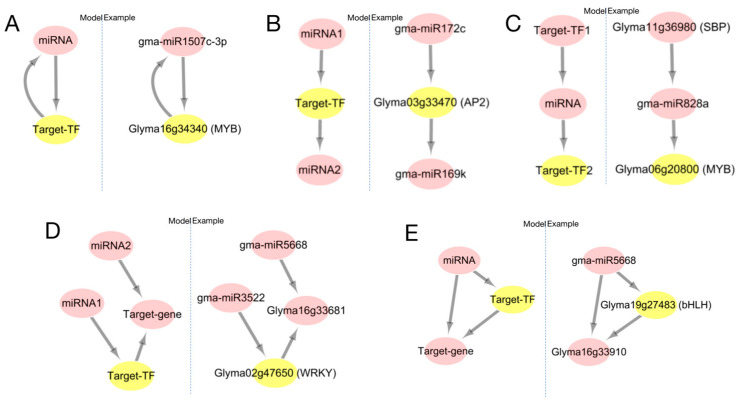
Five regulation patterns between miRNAs and transcription factors. (**A**) there is both positive and negative regulation between miRNA and target-TF; The arrows represent the target relationship ((**B**–**E**) are the same). (**B**) MiRNA1 regulates target-TF, while target-TF regulates miRNA2, completing the regulatory relationship between miRNA1 and miRNA2 through target-TF; (**C**) MiRNA is regulated by target-TF1, and the miRNA re-regulates target-TF2. The regulation between target-TF can be performed directly or be completed by miRNA; (**D**) MiRNA1 regulates target-TF, which regulates another gene. In addition, miRNA1 positively expresses the gene, while miRNA2 also regulates the negative expression of the gene. There are two expression patterns in this model; (**E**) MiRNA regulates both transcription factors and genes, and this transcription factor also regulates another gene.

Based on the analysis, we divided the mutual regulatory relationship between miRNA and target-TF into five patterns (Figure 10). Pattern one: there is both positive and negative regulation between the miRNA and target-TF (Figure 10A); pattern two: miRNA1 regulates target-TF, while target-TF regulates miRNA2, completing the regulatory relationship between miRNA1 and miRNA2 through target-TF (Figure 10B); pattern three: miRNA is regulated by target-TF1, and the miRNA re-regulates target-TF2. The regulation between target-TF can be performed directly or be completed by miRNA (Figure 10C); pattern four: miRNA1 regulates target-TF, which regulates another gene. In addition, miRNA1 positively expresses the gene, while miRNA2 also regulates the negative expression of the gene. There are two expression patterns in this model (Figure 10D); pattern five: miRNA regulates both transcription factors and genes, and this transcription factor also regulates another gene (Figure 10E). The interaction between these five miRNAs and TF also illustrates the expression of miRNA and TF (co- or reverse expression), which also explains the co-expression of miRNA and TF.

## 3. Discussion

MiRNAs are valuable tools for identifying potential disease resistance genes. By analyzing the pathways of identified genes and disease-resistant miRNAs, we can predict which genes may be related to disease resistance. In this paper, twelve genes were predicted (Figure 9), seven of which have been confirmed by related studies while five remain unreported. The five unreported genes could potentially be new resistance genes to *Phytophthora sojae*, providing a fresh perspective on the study of soybean *Phytophthora* resistance. Among them, Glyma17g183100 was found to be highly expressed in roots and nodules compared with other parts of the plant. The MYB-like DNA-binding protein was annotated as the homologous protein of the gene, which was consistent with the sequence of the 314-amino-acid FlbD protein. The FlbD protein had a transcriptional activation effect on the developmental regulatory gene brlA of the Aspergillus niger [30]. Both Glyma03g42310 and Glyma03g41420 were relatively highly expressed in leaves, with their gene atlas description co-expressed with genes in the leaf-specific co-expression subnetwork and roots-specific co-expression subnetwork, respectively. Glyma19g44520 is annotated as the ubiquitin-protein ligase SINAT5.

Since miRNA contains non-coding genes, collinear modules or the average Ks values of adjacent genes are used to calculate the differentiation time [31]. In 2011, the gene families of 12 homologous genomic regions of soybean were studied with three genome changes, resulting in the gene balance hypothesis being proposed [10]. The evolution model and co-evolution of miRNA genes and target genes were studied [32]. According to the above analysis (Figure 7), the miRNA-target was divided into five categories according to their Ks value, and it was found that the differential miRNAs were mainly concentrated in the miR394, miR393, miR166, and miR169 families. According to several miRNA production methods, we know that miRNA is produced by genes, and the Ks value of Gma-miR393d in Figure 7C was greater than the Glyma16g05500 Ks value, indicating that miRNA appeared earlier than Glyma16g05500. The inference that Gma-miR393d is produced by other genes with roles other than the regulation of *Phytophthora* root rot in soybean is consistent with what has been reported in these studies [33,34]. MiR393 is a gene found in Arabidopsis that regulates secondary metabolic pathways and may metabolize to produce compounds that more effectively inhibit biotrophic and semi-biotrophic pathogens [35]. Although different anti-microbial compounds were produced in soybean and Arabidopsis, it was found that the expression levels of GmHID1 and GmIFS1, which encode key enzymes in isoflavone biosynthesis, were significantly decreased in miR393 knockout soybean plants [36]. In addition to the gma-miR393 family, the gma-miR166 and miR169 families are also highly conserved sequences, which have important functions in organisms. It was found that the miR166 family can regulate biosynthesis and metabolism to control soybean plant height and shape [37]. In drought stress studies, both the miR166 and miR169 families played important regulatory roles [38]. These highly conserved miRNA families play a variety of roles in plant growth. In general, we found that the Ks of miRNA are more widely distributed than its target and occur in three evolutionary stages. When miRNA occurs earlier than its target, it indicates that miRNA production may originate from other genes. After target production, the miRNA acts on its target. Due to a weak effect or other reasons, new miRNAs are produced to coordinate regulation. With the production of miRNA-target, physiological strains of *Phytophthora* root rot will also evolve, at which time a new miRNA-target will be produced for regulation. The co-evolution of miRs and miRNA targets during soybean domestication is therefore proposed.

The family of transcription factors used in this study to search for binding sites included MYB, SBP, WRKY, AP2, ERF, and bHLH. These transcription factors have binding sites for proteins involved in miRNA formation, five verified soybean *P. sojae* resistant genes, and miRNAs corresponding to transcription factors. Studies have shown that miR828 regulates transcription factor MYB [39], miR4398 regulates transcription factor WRKY [40], and bHLH74 is regulated by miR396, controlling leaf development. miR396 is also involved in silting members of the transcription factor family GRF, regulating cell growth and leaf development [41]. It is also speculated that the WRKY gene also has action sites for miR396. There are several studies suggesting that miRNA regulates transcription factors. In recent years, elucidating the post-transcriptional regulation mechanism of miRNA genes has become the focus of miRNA function research. Binding sites on transcription factors and miRNA promoters have also been widely calculated and predicted [42]. Hackenberg et al. [32] used transgenic technology to prove that transcription factors in barley play an important regulatory role in the transcription of miRNA and other non-coding small RNAs. Through deep sequencing, they detected that miR156 showed up-regulated expression in transgenic plants. The up-regulated expression of miR168 in non-transgenic plants indicated that transcription factors also play an important role in the regulation of miRNA [43]. In this study, it was found that the regulatory relationship between miRNA and target-gene expression was not all reverse expression. For example, in Figure 10D, miRNA2 acts in reverse on the target gene while miRNA1 acts in reverse on target-TF, and target-TF acts in reverse on the target gene. In this mode, miRNA2 and the target gene are expressed in reverse. miRNA1 and the target gene are positively expressed through target-TF, which results in both reverse and forward expression levels between the miRNA-target. Therefore, the regulatory pattern of the miRNA target should be analyzed in combination with specific interaction networks.

## 4. Materials and Methods

### 4.1. Plant Materials and Planting

In this study, the Suinong No.10 soybean, the main cultivar in the second accumulated temperate zone of Heilongjiang Province, was selected for planting. The seeds were planted in a greenhouse at Northeast Agricultural University, Harbin, Heilongjiang, germinated, and grown in a vermiculite/pearl rock mixture (3:1 ratio) in a growth chamber. Sixteen-hour light and 8-h dark photo cycles were set at 23 °C in darkness and 26 °C in light with 80% humidity. The experiment included three sets of plants: control plants that were untreated, plants inoculated with carrot (CA) empty medium, and three plants inoculated with fungus. Each set was repeated three times.

### 4.2. Culture and Inoculation of the P. sojae

A carrot (CA) culture medium was used, the carrot was washed, chopped and juiced, and filtered using 8 layers of gauze. The filtrate was filled with ultra-pure water and sterilized at 121 °C for 20 min after adding agar, in which carrot:water:agar = 200 g:1 L:18 g. The sterilized medium was poured into a plate, cooled, and solidified. The fungus was placed in the center of the solid medium using a sterilized picking ring, and the side with the fungus was positioned next to the medium, sealed, and placed in a 30 °C incubator with no light for 10 days before inoculation. Under the same culture conditions, when the plant growth reached the V1 stage, the plants with similar growth were selected and inoculated by hypocotyl wound inoculation. A cut was made 1 cm below the opposite true leaves and the wound was about 1.5 cm long and 0.8 mm wide. A solid medium covered with fungus was inoculated on the wound and the wound was then wrapped with moist sterile cotton and cultured in a greenhouse at 25 °C.

### 4.3. Sample Acquisition and Gene Expression Profiling Sequencing

Leaf samples were taken at three time points, 0.5 h, 2 h, and 4 h after inoculation with *P. sojae*. The sampling sites consisted of three compound leaves. The three leaves were quickly removed, bagged, and placed in liquid nitrogen for instant cooling before being placed in a freezer for storage at −80 °C. All the RNA was extracted using a Qiagen RNeasy Mini Kit (Qiagen, Hilden, Germany) and sent to BGI for expression profile sequencing. The SCKL were the samples without any treatment. The SML were the samples inoculated with a CA empty medium. STL0.5 was sampled at 0.5 h inoculation with *P. sojae*. STL2 was sampled at 2 h inoculation with *P. sojae* and STL4 was sampled at 4 h inoculation with *P. sojae*. Gene expression after sequencing was represented by the RPKM value [44], which was calculated as follows:

(1) $$\mathrm{RPKM}=\frac{10^{6}C}{NL/10^{3}}$$

where *C* is the gene region read segment count, *N* is the sequencing depth, and *L* is the gene length.

### 4.4. Target Gene Prediction of miRNA

Initial studies on the mode of action of miRNA and target genes [45] showed that there was no vacancy in the mode of miRNA acting on target genes in plants, and a maximum of four mismatches. Later on, someone demonstrated that only one mismatch was allowed in the base region of position 212 except for the 10th and 11th bases [46]. psRNA target software, psRobot software, and BLAST local methods were used for prediction. According to the names of miRNA, its target genes, and the location of the miRNA’s target genes, three miRNA-target relationships predicted by the software were screened out. As miRNAs function through regulating gene expression [47], it can be considered that miRNAs regulating differentially expressed genes can respond to *P. sojae*. The related differentially expressed genes can be used as a *P. sojae* associated gene set. We look for their target genes in the gene concentration and then determine the miRNAs which are in response to *P. sojae*.

At the same time, to facilitate the subsequent analysis, Ensembl Plant database’s BioMart, local BLAST, and online BLAST in the Ensembl Plant database were found to correspond with the GenBank ID in the sequencing result as well as the Gene stable ID (V1.0). The sequence described by GenBank ID was compared with all genes in the soybean genome V1.0 (JGI-Glyma-1.1) version for homologous sequence comparison, to assess whether the location data was consistent. The match with the highest score was finally selected as the conversion result. The conversion results are presented in Table S9.

### 4.5. Quantitative Real-Time PCR(qRT-PCR)

TRIzol reagent (Invitgen, Carlsad, CA, USA, Cat no. 15596-026) was used to extract the total RNA [48]. For the synthesis of cDNAs for the set of genes associated with the response to *P. sojae*, we added 4 μL 5* Prime Script Buffer, 1 μL Oligo (dT) (500 μg/mL), 1μLRandom6mers,1 μL Prime Script RTEnzyme Mix, 3 μL Total RNA (1 ng–5 μg), and 10 μL RNase Free dH2O reagents to a 0.2 mL Eppendorf tube of RNaseree. We gently mixed each component uniformly, incubated at 37 °C for 2 min, and then placed it into a PCR instrument, incubated at 37 °C for 50 min, and heated it at 70 °C for 15 min to abort the reaction. It was stored at −20 °C.

The creation of particular stem-loop primers, which are made up of universal stem-loop and miRNA-specific sequences, is necessary for the reverse transcription of miRNA. Zhao et al. [49] created the universal stem ring using the sequence CTCAA CTGGT GTCGT GGAGT CGGCA ATTCA GTTGA G. This technique has great specificity, since the miRNA-specific sequence is a 7–8 nucleotide sequence that is complementary to the 3′ end of the miRNA (Supplementary Materials Table S10). dsDNase (Code R2028 US; Everbright Corporation, Suzhou, China) and Fast Super EvaGreen qPCR Master Mix were used to create the UEIris II RT-PCR system for the first gene strand of miRNA cDNA responding to Botrytis cinerea root rot (Code S2008 US; Everbright Corporation).

As a downstream primer for qRT-PCR, a section of the sequence 5′-TGGTG TCGTG GAGTC G-3′ was chosen, because it is complementary to a known miRNA-specific stem-loop primer sequence. The upstream primer should be a section of fixed sequence ACACT CCAGC TGGG+1118 nucleotide sequences complementary to the 5′ end of miRNA, according to the design concept of Zhao et al. [49] (Supplementary Materials Table S11).

The cDNA was diluted step-by-step 10, 100, 1000, 10,000, and 100,000 times for the primer amplification efficiency assay, and the PCR system was followed by the qRT-PCR detailed in the next section. The results were collated to plot the primer amplification efficiency curve (Supplementary Materials Figure S1). The primer amplification efficiencies calculated from the slope and R2 values were all in the range of 0.9–1.1, meeting the experimental requirements. After qRT-PCR amplification, the specific amplification was evaluated according to the melting curve and amplification curve. The relative expression levels were analyzed by applying the ΔΔCT (formula) method in the Rotor-Gene Q Series 1.7 Software. Actin [50] and F-BOX [51,52,53] were used as reference genes of target genes and miRNA genes for the relative quantification analysis of the expression levels of target genes. All experiments were repeated three times. ΔΔCT calculation formula is as follows:

ΔCT(test) = CT(target, test) − CT(ref, test) ΔCT(calibrator) = CT(target, calibrator) − CT(ref, calibrator) ΔΔCT = ΔCT(test) − ΔCT(calibrator)(2)

### 4.6. Target Gene Pathway Analysis and Functional Annotation

To construct the wizard tree for miRNAs and target genes, sequence similarity was analyzed separately. First, mature miRNAs were compared to their target genes using clustalx software (V 2.0) [54] for multiple sequence comparisons. Then, mega-X software (V 10.1.7) [55] was used to automatically generate the wizard tree based on these results [55]. Since mature miRNAs are characterized by short sequences and high similarity, and the classification of miRNA families is based on sequence similarity, mature miRNAs were classified into miRNA families for this study. Furthermore, the specificity of miRNAs was classified by querying the miRBase for each species of included miRNAs, and the clustering results were statistically analyzed. The miRNA 555 sequences were downloaded from miRBase version (20) (Supplementary Materials Table S12). Firstly, the miRNA-target relationships were analyzed, then classified, and the function of certain classes of miRNAs could be predicted.

The Gene Ontology (GO) database (http://www.geneontology.org, accessed on 26 July 2022)) and the Kyoto Encyclopedia of Genes and Genomes (KEGG) database (http://www.genome.jp/kegg/mapper.html, accessed on 26 July 2022) were used for gene annotation. Using hypergeometric tests, genes in the entire genome were set as the background to identify pathways significantly enriched in candidate target genes [54]. The relationship between miRNA-target and pathway annotation information was presented using Cytoscape software (V 3.8.0). The annotation information of genes provided by expression profiling data, the classification of miRNA-target relationships obtained by clustering analysis, and the plant-pathogen pathway closely related to disease resistance were obtained using Cytoscape software (V 3.8.0). The information of miRNAs, targets and their annotation in the plant–pathogen pathway was presented. The functions of the miRNAs were inferred from the functions of the target genes. This, combined with the current understanding of the plant–pathogen pathways roughly signifies the location where miRNAs play a role in the pathway.

### 4.7. Prediction of miRNA and Transcription Factor Regulation, and Analysis of Interaction Patterns

Entire soybean transcription factors were downloaded from the PlantTFDB database [55] and intersected with RAPG, where a cluster analysis was performed to obtain the transcription factors in response to *P. sojae*. Several proteins are involved in plant miRNA formation, among which DCL1 is involved in the formation of the vast majority of miRNAs in Arabidopsis; SE proteins may be implicated in the formation process from pri-miRNA to pre-miRNA; HASTY proteins are involved in miRNA transport, transporting miRNA duplexes to the cytoplasm; HEN1 is involved in the methylation of the nucleic acid 2 hydroxyl group at the 3′ end of double-stranded miRNA, that is, in the formation of stable structures by miRNA; HYL 1, also called DRB1, is a protein that interacts with DCL1, while the last protein, the AGO protein, is an essential component of the RISC complex and is also the most diverse [56,57]. Searching in the NCBI GENE database using protein names and links related to soybean, or related to plants, were selected to obtain gene-positional data.

If the gene is on the positive strand of the genome, the promoter sequence will be between (start −2000 bp, start) and if the gene is on the negative strand of the genome, the promoter position will be between (end + 2000 bp, end). The promoter sequence is then taken. MiRNA-regulating transcription factors were selected to obtain information from the miRBase database about the location of miRNA on the genome and promoter sequences using the Map Viewer tool. If the annotation information in the miRBase shows that the miRNA position is on the negative strand, select the plus strand (start −2000 bp, start) as the promoter sequence in Map Viewer, and select the minus strand (end, end + 2000 bp) as the promoter sequence in Map Viewer if the annotation information in the miRBase shows that the miRNA position is on the positive strand.

Using JASPAR2022 (https://jaspar.genereg.net/, accessed on 26 July 2022) for prediction, 27 families of plant transcription factors related to MYB, SBP, WRKY, AP2, ERF, and bHLH were selected for one-to-one prediction with promoters and results with a score greater than 10 were selected as transcription factor binding sites.

### 4.8. Analysis of miRNA and Target Gene’s Evolution

MiRNAs function by regulating target genes and miRNA evolution is correlated with the evolution of genes. MiRNA production can result from several modalities: whole genome duplication (WGD), large-scale duplication (LSD), and tandem duplication (TD). Large segmental duplications can be either forward or reverse. The time of evolutionary occurrence is judged by the *Ks* value method, but as miRNAs are non-coding small RNAs, the *Ks* value cannot be calculated directly and can be used to encode proteins on the module where they are located. The average *Ks* value of a gene indicates the *Ks* value of a miRNA gene. The average *Ks* value of the coding genes in each module can be used to calculate the doubling time T, calculated as:

(3) $$T=\frac{Ks}{2E}$$

where *E* represents the molecular replacement rate.

The divergence time of miRNAs can be calculated via the average Ks value of the protein-coding genes on the module. Therefore, exploring the evolutionary situation of conserved miRNA is of great significance to the study of the evolution of organisms [58]. Fossil evidence as well as a comparison of molecular evolution analyses show that soybean has a molecular replacement rate one-fifth that of Arabidopsis, with a synonymous substitution rate of 1.5 × 10^−8^ for Arabidopsis, and 6.1 × 10^−9^ for soybean.

## Data Availability

The data of the Suinong No.10 soybean line expression profile can be found here: https://github.com/Jinsong1999/All_Data, accessed on 1 February 2023.

## Supplementary Materials

The supporting information can be downloaded at: https://www.mdpi.com/article/10.3390/ijms24108546/s1.
